# Multifocal Transcranial Direct Current Stimulation Modulates Resting-State Functional Connectivity in Older Adults Depending on the Induced Current Density

**DOI:** 10.3389/fnagi.2021.725013

**Published:** 2021-11-26

**Authors:** Kilian Abellaneda-Pérez, Lídia Vaqué-Alcázar, Ruben Perellón-Alfonso, Cristina Solé-Padullés, Núria Bargalló, Ricardo Salvador, Giulio Ruffini, Michael A. Nitsche, Alvaro Pascual-Leone, David Bartrés-Faz

**Affiliations:** ^1^Department of Medicine, Faculty of Medicine and Health Sciences, Institute of Neurosciences, University of Barcelona, Barcelona, Spain; ^2^Institute of Biomedical Research August Pi i Sunyer (IDIBAPS), Barcelona, Spain; ^3^Section of Neuroradiology, Department of Radiology, Diagnostic Image Center, Hospital Clinic of Barcelona, University of Barcelona, Barcelona, Spain; ^4^Magnetic Resonance Image Core Facility (IDIBAPS), Barcelona, Spain; ^5^Neuroelectrics, Cambridge, MA, United States; ^6^Neuroelectrics, Barcelona, Spain; ^7^Leibniz Research Centre for Working Environment and Human Factors, Dortmund, Germany; ^8^Department of Neurology, University Medical Hospital Bergmannsheil, Bochum, Germany; ^9^Hinda and Arthur Marcus Institute for Aging Research and Deanna and Sidney Wolk Center for Memory Health, Hebrew SeniorLife, Boston, MA, United States; ^10^Department of Neurology, Harvard Medical School, Boston, MA, United States; ^11^Guttmann Brain Health Institute, Guttmann University Institute of Neurorehabilitation, Autonomous University of Barcelona, Badalona, Spain

**Keywords:** aging, electric current density, multifocal transcranial direct current stimulation, resting-state functional magnetic resonance imaging, non-invasive brain stimulation (NIBS), electric modeling, neuroimaging

## Abstract

Combining non-invasive brain stimulation (NIBS) with resting-state functional magnetic resonance imaging (rs-fMRI) is a promising approach to characterize and potentially optimize the brain networks subtending cognition that changes as a function of age. However, whether multifocal NIBS approaches are able to modulate rs-fMRI brain dynamics in aged populations, and if these NIBS-induced changes are consistent with the simulated electric current distribution on the brain remains largely unknown. In the present investigation, thirty-one cognitively healthy older adults underwent two different multifocal real transcranial direct current stimulation (tDCS) conditions (C1 and C2) and a sham condition in a crossover design during a rs-fMRI acquisition. The real tDCS conditions were designed to electrically induce two distinct complex neural patterns, either targeting generalized frontoparietal cortical overactivity (C1) or a detachment between the frontal areas and the posteromedial cortex (C2). Data revealed that the two tDCS conditions modulated rs-fMRI differently. C1 increased the coactivation of multiple functional couplings as compared to sham, while a smaller number of connections increased in C1 as compared to C2. At the group level, C1-induced changes were topographically consistent with the calculated electric current density distribution. At the individual level, the extent of tDCS-induced rs-fMRI modulation in C1 was related with the magnitude of the simulated electric current density estimates. These results highlight that multifocal tDCS procedures can effectively change rs-fMRI neural functioning in advancing age, being the induced modulation consistent with the spatial distribution of the simulated electric current on the brain. Moreover, our data supports that individually tailoring NIBS-based interventions grounded on subject-specific structural data might be crucial to increase tDCS potential in future studies amongst older adults.

## Introduction

The human brain is organized into complex neural networks that can be studied through resting-state functional magnetic resonance imaging (rs-fMRI; Damoiseaux et al., 2006; Smith et al., 2009; van den Heuvel and Sporns, 2013; Petersen and Sporns, 2015). Some of these neural systems have been shown to support cognition (Bressler and Menon, 2010) and change through the lifespan (Dosenbach et al., 2010; Betzel et al., 2014), as well as to be highly susceptible to aging (Tomasi and Volkow, 2012; Ferreira and Busatto, 2013; Sala-Llonch et al., 2015; Nashiro et al., 2017).

Non-invasive brain stimulation (NIBS) protocols have been used to modulate these functional systems subtending cognition in older adults (Abellaneda-Pérez et al., 2019a). The combination of NIBS with neuroimaging techniques in these populations has shed light into the network plasticity mechanisms underlying cognitive aging (i.e., Abellaneda-Pérez et al., 2019b) as well as on the putative neurobiological mechanisms underlying NIBS-induced phenotypic improvements in the elderly (i.e., Holland et al., 2011; Meinzer et al., 2013; Antonenko et al., 2018; Nilakantan et al., 2019).

In the latest years, transcranial direct current stimulation (tDCS) has been widely employed, particularly in advancing age (Perceval et al., 2016; Tatti et al., 2016). In conventional tDCS studies, which aim to target discrete cortical regions, a single anode accompanied by its corresponding cathode is used. According to our current mechanistic understanding, during tDCS, neural membrane potentials are depolarized under the anode, leading to an increase in cortical excitability, while they are hyperpolarized under the cathode, thus diminishing cortical excitability at the macroscopic level (Nitsche and Paulus, 2000; Nitsche et al., 2008). More recently, novel multifocal or network-based tDCS protocols have been developed in order to target multiple brain areas simultaneously (Ruffini et al., 2014). In multifocal tDCS, multiple electrodes with differential intensities and polarities are employed such that the resulting field aims to maximally target a specific distributed brain network and can result in higher modulatory efficacy than an otherwise similar two-electrode tDCS approach in younger samples (Fischer et al., 2017). However, in what manner multifocal tDCS entails capability to influence rs-fMRI dynamics in the aging brain, and whether this modulation is consistent with a previously targeted neural pattern, has not been previously investigated, despite the potential of network-based approaches to directly modulate a complex system rather than a concrete region. In this vein, a whole network modulation is particularly relevant in aging, since, as observed in numerous descriptive fMRI investigations, brain networks are less integrated and more segregated as a function of age (Cao et al., 2014; Chan et al., 2014; Sala-Llonch et al., 2014; Grady et al., 2016; Spreng et al., 2016), probably reflecting age-associated dedifferentiation processes (Park et al., 2012). Notably, it has been proposed within the cognitive neuroscience of aging literature that this loss of brain networks integration in the elderly relates to poorer cognitive performance (i.e., Sala-Llonch et al., 2014; Vidal-Piñeiro et al., 2014). Consequently, altering multiple neural nodes within or between specific neural circuits based on a previously simulated electrical pattern on the brain could be used to potentially restore a regular brain functioning and thus, hypothetically, ameliorate cognitive decline in advanced age.

The present study leverages from a previous investigation by our group and is based on its fMRI findings. In the stated report, we observed that distinct groups of older adults, with comparable educational attainment but different levels of white matter hyperintensities burden, can engage dissimilar brain activity patterns to successfully solve a particular working memory paradigm (for further details on the original brain patterns, see Fernández-Cabello et al., 2016). In the present study, two real tDCS conditions were designed based on those previous fMRI findings to induce two distinct electrical distribution configurations, which were expected to produce either generalized frontoparietal cortical overactivity (i.e., condition 1, or C1) or an antero-posterior dissociation aiming to enhance frontal areas whereas reducing the posteromedial cortex activity (i.e., condition 2, or C2; see section “tDCS parameters” for detailed information). It is worth noting that there is a correspondence between those brain functional configurations observed during activation and at rest (Smith et al., 2009). Hence, while C1 patterns are consistent with the classical frontoparietal circuits observed in rs-fMRI studies, the C2 anatomically resemble both the frontoparietal as well as the default-mode networks (see also Abellaneda-Pérez et al., 2020). The goals of the present study were: (I) to explore the impact of two distinct multifocal tDCS montages on rs-fMRI associated connectivity changes in older adults; (II) to examine the topographical correspondence between the tDCS-induced rs-fMRI effects and the simulated electric current distribution on the brain at the group level; and (III) to individually determine whether, and how, the observed rs-fMRI changes are associated with the calculated electric current values.

## Materials and Methods

### Participants

As in our previous studies (Vidal-Piñeiro et al., 2014; Vaqué-Alcázar et al., 2017, 2020), subjects participating in the present investigation were recruited from the Fundació Institut Català de l’Envelliment. We contacted thirty-seven subjects and initially included thirty-three participants fulfilling the following criteria: aged older than 65 and neuropsychological assessment within range of normality (see below). Selected exclusion criteria were Hamilton Depression Rating Scale > 13, history of epilepsy, neurological or psychiatric disorders and any NIBS-related contraindication (Rossi et al., 2009, 2021; Antal et al., 2017) as well as for the MRI. One subject was discarded for a previous single episode of absence seizure and another volunteer was excluded due to morphine pump implantation to treat chronic pain.

Finally, thirty-one subjects met criteria to participate in this study. All participants were tDCS naïve and right-handed older adults [mean age ± standard deviation (*SD*), 71.68 ± 2.5 years; age range, 68 – 77 years; 19 females; years of education mean ± *SD*, 12.29 ± 4.0 years]. All volunteers provided informed consent in accordance with the Declaration of Helsinki (1964, last revision 2013). All study procedures were approved by the Institutional Review Board (IRB 00003099) at the University of Barcelona. For all participants, MRI images were examined by a senior neuroradiologist for any clinically significant pathology (none found).

### Experimental Design

**FIGURE 1:**
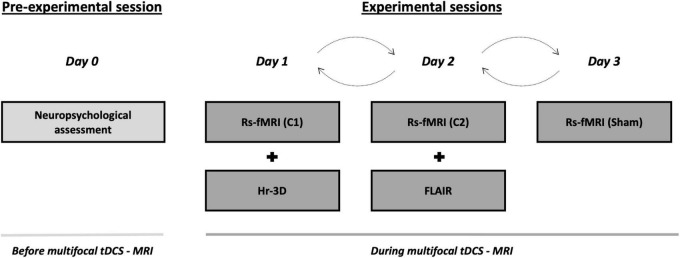
Study protocol and timeline of the procedures accomplished before and during multifocal tDCS-MRI. On the pre-experimental session, all the subjects undertook a neuropsychological screening session to verify a normal cognitive functioning. Then, during the three consecutive experimental sessions, transcranial stimulation was delivered using differential tDCS montages (i.e., C1, C2 and sham) in a counterbalanced fashion while rs-fMRI data was acquired. A hr-3D and a FLAIR datasets were acquired for additional examinations. tDCS, transcranial direct current stimulation; MRI, magnetic resonance imaging; rs-fMRI, resting-state functional MRI; hr-3D, high-resolution three-dimensional; FLAIR, fluid-attenuated inversion recovery; C1, condition 1; C2, condition 2.

The present study was conducted in a randomized single-blind sham-controlled crossover design that consisted of four visits to our center. On the first visit (i.e., pre-experimental session; day 0), all participants underwent a comprehensive neuropsychological assessment to ensure cognitive functioning within the normal range according to age and years of education (see section “Neuropsychological assessment”). Subsequently, three experimental sessions with distinct multifocal tDCS conditions (days 1, 2, and 3) were conducted while acquiring MRI data during brain stimulation. These MRI acquisitions comprised, firstly, an arterial spin labeling (ASL) dataset (∼ 6 min), secondly, an rs-fMRI acquisition (∼ 8 min), and finally, a task-based fMRI dataset (∼ 11 min). The present study focuses on rs-fMRI images to investigate in which manner the simulated electric current distributions are associated with the actual experimentally induced effects on the aged brain when not engaged in any particular task. Furthermore, on day 1, a high-resolution three-dimensional (hr-3D; ∼ 8 min) dataset was acquired for functional data preprocessing purposes and to estimate the distribution of the induced electric current. In addition, the mentioned hr-3D acquired on day 1, and a fluid-attenuated inversion recovery (FLAIR) sequence obtained on day 2 (∼ 3 min), were used for neuroradiological assessment to exclude any brain structural abnormalities in study participants. Finally, a diffusion tensor imaging was obtained on day 3 (∼ 9 min; not used in this investigation). Therefore, the total duration of the experimental sessions was ∼ 40 min, whereof the first ∼ 5 min were used for tDCS-MRI setting preparation, and the remaining ∼ 35 min were entirely conducted within the MRI scanner. Out of the total in-scanner time, the initial 25 min were acquired during brain stimulation, and the final variable minutes were dedicated to additional acquisitions. In the experimental sessions, multifocal tDCS was applied to all participants using the two different montages referred above (i.e., C1 and C2) with real stimulation as well as a sham stimulation condition (see section “tDCS parameters” for further details). The order of the experimental sessions was counterbalanced. There was a minimum one-month wash-out between each experimental session to avoid possible prevalence of after-effects (Figure 1). Further, a questionnaire of tDCS-related adverse events was administered at the end of each experimental session and compared between the experimental conditions [all p-values > 0.05; for further details see Supplementary Table 1 and Supplementary Material]. In addition, a very brief assessment of the quality of sham was administered (for further details, see Supplementary Material).

### Neuropsychological Assessment

A comprehensive battery of neuropsychological tests covering all cognitive domains was administered, including the Rey-Osterrieth Complex Figure (ROCF), Rey Auditory Verbal Learning Test (RAVLT), Boston Naming Test (BNT), Semantic category evocation of animals, Number location and incomplete letters from the Visual Object and Space Perception Battery (VOSP), Trail Making Test (TMT), parts A and B, Phonemic fluency (FAS), Stroop Color Word Test, Symbol Digit Modalities Test (SDMT), and Digit span forward and backward from WAIS-III. Finally, the Vocabulary Subtest from WAIS-III was also administered to have a measure of premorbid intelligence. All participants presented a normal cognitive profile with mini-mental state examination (MMSE) scores of ≥27 and performance scores not more than 1.5 *SD* below normative data (adjusted for age and years of education) on any of the administered neuropsychological tests (i.e., they did not fulfill the criteria for mild cognitive impairment; Petersen and Morris, 2005).

### Transcranial Direct Current Stimulation Parameters

**FIGURE 2:**
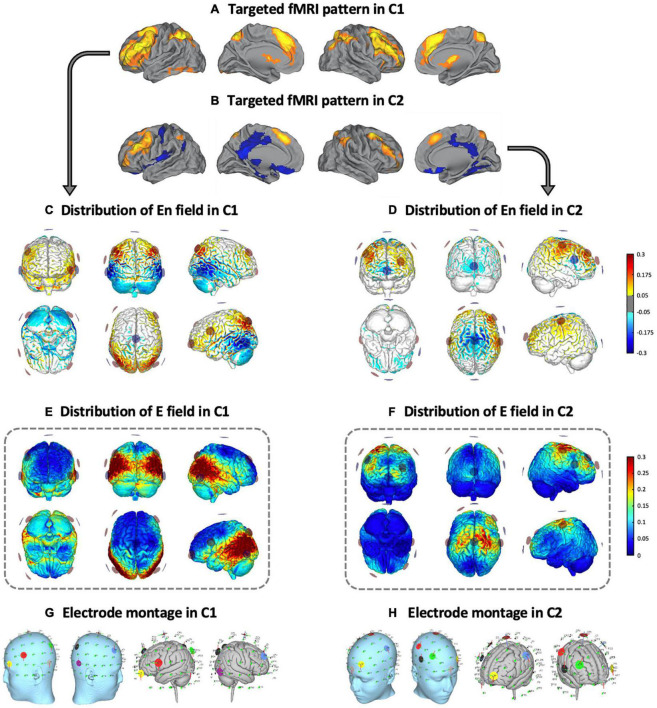
Multifocal tDCS montages. Targeted fMRI patterns in **(A)** C1 and **(B)** C2, from Fernández-Cabello et al. (2016, adapted with permission). Distribution of the *E*_*n*_ field in the cortical surface in **(C)** C1 and **(D)** C2 (in V/m units). Distribution of the electric field’s magnitude in the cortical surface in **(E)** C1 and **(F)** C2 (in V/m units). Representation of the electrode locations and shape on the scalp (left) and the gray matter (right) based on a Montreal Neurological Institute (MNI) head in **(G)** C1 and **(H)** C2, obtained for visual purposes. fMRI, functional magnetic resonance imaging; C1, condition 1; C2, condition 2.

Two distinct multifocal tDCS montages were designed with the Stimweaver montage optimization algorithm (Ruffini et al., 2014). The latter determines the positions and currents of the electrodes over the scalp that induce an electric field in the brain that better approximates a weighted target electric field map. We optimized for the electric field component normal (orthogonal, *E_n_*) to the cortical surface, assuming a first order model for the interaction of the electric field with neurons in the cortex: when *E_n_* points into/out of the cortical surface (positive/negative values of *E_n_* in our convention), this leads to an increase/decrease in the membrane potential of the soma of pyramidal cells (and hence, cortical excitability). As mentioned, the weighted target *E_n_*-maps used in this study were designed based on the findings obtained in our previous fMRI study (Fernández-Cabello et al., 2016). More precisely, on the one hand, the C1 montage was grounded on an fMRI pattern of extended cortical activity, including the bilateral middle frontal gyri, the paracingulate gyri, the precuneus cortex, the bilateral supramarginal gyri and/or intraparietal sulcus area and the lingual gyri (Figure 2A). On the other hand, the C2 montage was derived from a second fMRI pattern including moderate activity increases in the bilateral middle frontal and the paracingulate gyri, altogether with brain activity decreases of the posterior cingulate gyrus, the ventral precuneus, and the precentral gyri (Figure 2B). In the optimization, the regions that registered a brain activity increase/decrease were targeted with an excitatory/inhibitory (positive/negative) target *E_n_* field (see Figure 2C for C1, and Figure 2D for C2). Cortical maps of the magnitude of the induced electric field, in which we focused in this investigation, are shown framed in Figure 2E for C1 and in Figure 2F for C2, in the template head model used in the optimization (Colin27; Miranda et al., 2013). Stimulation was delivered via an MRI-compatible Starstim Neuroelectrics device, using 8 circular MRI Sponstim electrodes with an area of 8 cm^2^. These MRI-compatible electrodes consist of a carbon rubber core and a sponger cover, both translucid materials. Hence, the core electrodes were located on the participant’s scalp by fitting them inside the sponge and into the holes of a neoprene cap corresponding to the 10/10 international system for electrode placement. The central Cz position was aligned to the vertex of the head in every subject to ensure an accurate cap placement.

Based upon the foregoing, in the C1 montage the electrodes were placed in the AF7, F4, FC5, P3, P4, P7, P8 and Cz positions (see Figure 2G and Table 1), while in the C2 montage the electrodes were placed in the AF3, C3, C4, F4, FC6, Fpz, Oz and Cz positions (see Figure 2H and Table 1). For sham stimulation, either C1 or C2 montages were randomly used. The stimulator was situated outside the MRI room and the electrodes were soaked with saline solution and a thin layer of Ten20 conductive paste to ensure good conductivity and stability throughout the MRI acquisition. The current was delivered to each electrode with a wireless neurostimulator (Starstim, by Neuroelectrics Barcelona) connected to a computer via Bluetooth. For safety issues, the maximum current delivered by any electrode was 2 mA, while the maximum current injected through all the electrodes was 4 mA. In the real intervention conditions, the current was supplied during the whole rs-fMRI acquisition, starting just before the ASL acquisition and concluding at the end of the task-based fMRI dataset. In all groups, the current was initially increased and finally decreased in a 30 s ramp-up and ramp-down fashion, carefully configured to not overlap with the MRI acquisitions. For the sham condition, the current dosage was composed of an initial ramp-up of 30 s immediately followed by a 1 min ramp-down, and a final ramp-down of 30 s immediately preceded by a ramp-up of 1 min.

**TABLE 1:** Electrode positions and current intensities in C1 and C2.

C1	C2
AF7: 363 μA	AF3: 864 μA
F4: 539 μA	C3: 665 μA
FC5: 385 μA	C4: 1,291 μA
P3: 1,493 μA	F4: 1,179 μA
P4: 1,220 μA	FC6: −966 μA
P7: −1,705 μA	Fpz: −998 μA
P8: −1,525 μA	Oz: −396 μA
Cz: −770 μA	Cz: −1,639 μA

### Magnetic Resonance Imaging Acquisition

All participants were scanned with a Siemens Magnetom Trio Tim Syngo 3 Tesla system at the MRI Core Facility (IDIBAPS) of the Hospital Clinic of Barcelona, Barcelona, Spain. In the succeeding, only the acquisitions used in this investigation, both for research purposes and clinical screening, are detailed. Three identical rs-fMRI datasets [T2*-weighted GE-EPI sequence; interleaved acquisition; repetition time (TR) = 2,700 ms; echo time (TE) = 30 ms; 40 slices per volume; slice thickness = 3.0 mm; interslice gap = 15%; voxel size = 3.0 x 3.0 x 3.0 mm; field of view (FOV) = 216 mm; 178 volumes] were acquired, one each experimental day. Furthermore, a hr-3D structural dataset [T1-weighted magnetization-prepared rapid gradient-echo (T1-weighted MPRAGE); sagittal plane acquisition; TR = 2,300 ms; TE = 2.98 ms, inversion time (IT) = 900 ms; slice thickness = 1.0 mm; voxel size = 1.0 x 1.0 x 1.0 mm; FOV = 256 mm; 240 slices] was acquired on experimental day 1. In addition, an axial FLAIR sequence (TR = 9,000 ms; TE = 96 ms; slice thickness = 3.0 mm; FOV = 240 mm; 40 slices) was obtained on experimental day 2 (see Figure 1).

### Functional Connectivity Analyses

The FMRIB Software Library (FSL; version 6.00^1^) and the Analysis of Functional NeuroImages (AFNI^2^) were used for preprocessing and analyzing functional neuroimaging data.

#### Functional Connectivity Preprocessing

Resting-state functional magnetic resonance imaging (rs-fMRI) data preprocessing included the removal of the first five volumes, motion correction, skull stripping, spatial smoothing [Full Width at Half Maximum (FWHM) = 7 mm], grand mean scaling and filtering with both high-pass and low-pass filters (0.01- and 0.1-Hz thresholds, respectively). Data were then regressed with six rigid-body realignment motion parameters, mean white matter, and mean cerebrospinal fluid signal. No global signal regression was used. Normalization to MNI standard space was also applied. A visual inspection of the preprocessed rs-fMRI images was thoughtfully completed before conducting further functional connectivity analyses. Moreover, as head movement may affect rs-fMRI results (Power et al., 2012, 2015; Van Dijk et al., 2012), in-scanner head motion was calculated for every subject. More precisely, two standard measures to estimate in-scanner head motion were obtained in a similar manner as described elsewhere (Power et al., 2012). Displacement relative to a single reference volume (absolute displacement) and relative to the precedent volume (relative displacement) were calculated for every subject. In our sample, no significant differences were found between the three conditions (i.e., C1, C2 and sham), considering both absolute and relative displacement (all p-values > 0.05; for further data, see Supplementary Table 2).

#### Regions of Interest-Based Functional Connectivity Analyses

Functional connectivity analyses were implemented based on a whole-brain atlas that parcels the brain into a set of anatomical regions of interest (ROIs). The selected atlas was the one developed for the CONN toolbox (Whitfield-Gabrieli and Nieto-Castanon, 2012). This atlas includes a rich set of regions to perform comprehensive whole-brain analyses using ROI-based approaches. More specifically, this atlas includes 132 ROIs, combining the FSL Harvard-Oxford cortical (91 ROIs) and subcortical atlases (15 ROIs) and the cerebellar areas from the Anatomical Automatic Labeling (AAL) atlas (26 ROIs). Individualized time-series of the different ROIs were extracted from the preprocessed and regressed images. In order to obtain a resting-state functional connectivity (rs-FC) measure for each ROI-to-ROI connection in each subject, the acquired ROI time-series were correlated with one another to create correlation matrices, using Pearson product-moment correlations.

### Electric Current Computations

SimNIBS 3.0.7 was used to individually calculate the electrical current induced by tDCS based on the finite element method (FEM) and individualized head models derived from the structural MRI datasets (^3^ Windhoff et al., 2013; Thielscher et al., 2015). First, T1-weighted anatomical images were used to create individualized tetrahedral FE head meshes of each subject, using MATLAB toolboxes (Nielsen et al., 2018), MeshFix (Attene, 2010), and Gmsh (Geuzaine and Remacle, 2009). These head models contain representations of the scalp, skull, cerebrospinal fluid (including the ventricles), eyeballs, gray-matter and white-matter. Second, the electrode positions (i.e., the center coordinates of the modeled electrodes) were placed on each subject head mesh, according to the locations established for each montage (i.e., C1 and C2). Then, electric current simulations were computed for each condition separately. Following the specific characteristics of the MRI Sponstim electrodes from Neuroelectrics, the electrode shape was set as elliptical, and the size was defined as 2.3 cm of diameter and 1 mm of thickness. The electrode’s sponge size was defined as 3.2 cm of diameter and 3 mm of thickness. Tissue and electrode conductivity values were set as default in SimNIBS software (Thielscher et al., 2011; Saturnino et al., 2015). Third, individual results were averaged together for each condition, resulting in group averages and *SD*s of the electric current density distribution. Finally, for each montage, data on the peak fields (99.9th percentile) and the electric current magnitude values within the selected ROIs, based on fMRI findings, were obtained (see sections “Statistical analyses” and “Multifocal tDCS effects on rs-fMR” for further information). The magnitude of the current density (normJ) was used in all the subject-based analyses of this investigation (i.e., those computed with SimNIBS). Current density seems to be particularly useful for dosage determination in terms of the regional quantity of current reaching the brain with advancing age (i.e., Indahlastari et al., 2020). Hence, if not otherwise specified (i.e., as in the originally designed models using a template brain), figures and analysis results reflect the electric current density in all cases, and are expressed as amperes per square meter (A/m^2^). Due to technical issues during the generation of the head mesh, related to poor quality of the T1 MRI data, a subject was discarded from these analyses. The segmentations quality from all generated brain images was individually examined and deemed appropriate.

### Statistical Analyses

Data analyses were performed using IBM SPSS (IBM Corp. Released 2017. IBM SPSS Statistics for Windows, Version 25.0. Armonk, NY: IBM Corp.) and MATLAB (Version R2019a, The MathWorks Inc., Natick, MA, United States).

#### Functional Connectivity Statistical Analyses

Resting-state functional connectivity (rs-FC) correlation matrices, permutation testing and pixel correction for multiple comparisons were performed using custom made MATLAB scripts. Functional connectivity differences were compared between conditions using non-parametric permutation testing (Nichols and Holmes, 2002). We chose this method because it does not rely on assumptions about the distribution of the data and correction methods for multiple comparisons can be easily implemented (Theiler et al., 1992).

**FIGURE 3:**
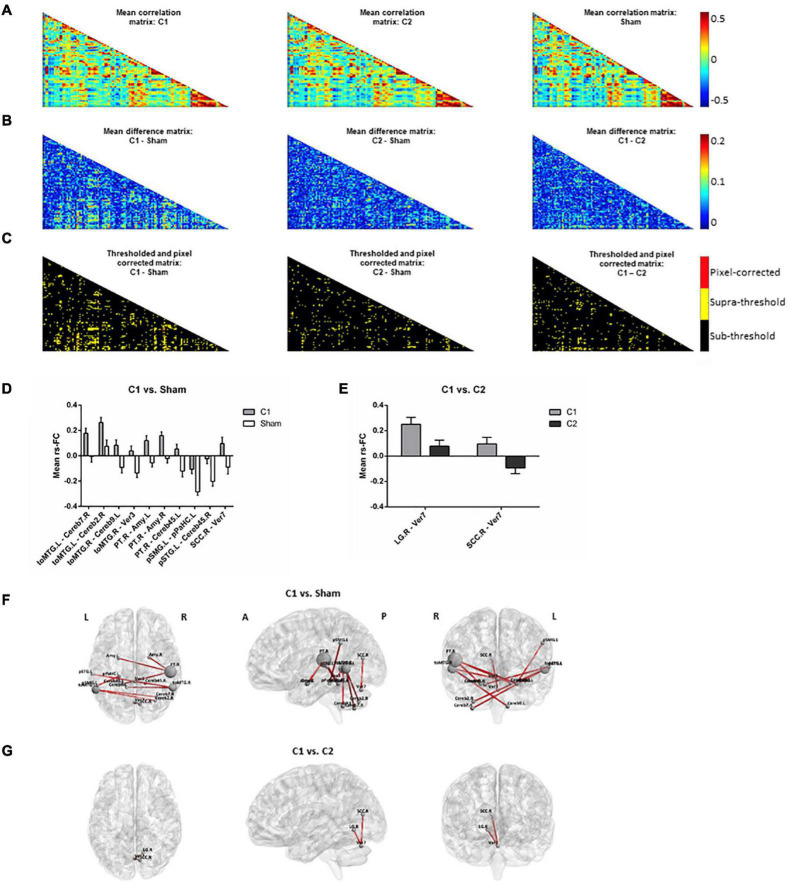
Rs-fMRI analyses. **(A)** Mean correlation matrices for (left to right): C1, C2 and sham. **(B)** Mean difference matrices for (left to right): C1 – Sham, C2 – Sham, C1 – C2. **(C)** Thresholded and pixel-corrected matrices for (left to right): C1 – Sham, C2 – Sham, C1 – C2 (dark: sub-threshold; yellow: supra-threshold; red: pixel-corrected). Bar-plots showing the mean with standard error of the mean (*SEM*) of rs-FC comparing **(D)** C1 vs. Sham and **(E)** C1 vs. C2, computed for visually inspecting the group tendencies in each coupling. Representation of the significant connections for **(F)** C1 vs. Sham and **(G)** C1 vs. C2 on a standard map (left to right: axial, sagittal and coronal view). Note that in Panel **(F)**, selected main hubs (i.e., those ROIs entailing ≥2 significant rs-FC connections when comparing C1 vs. Sham) are displayed with a correspondingly larger size to emphasize them. C1, condition 1; C2, condition 2; toMTG, Middle temporal gyrus, temporo-occipital part; Cereb, Cerebellum; Ver, Vermis; PT, Planum temporale; Amy, Amygdala; pSMG, Supramarginal gyrus, posterior division; pPaHC, Parahippocampal gyrus, posterior division, pSTG, Superior temporal gyrus, posterior division; SCC, Supracalcarine cortex; LG, Lingual gyrus; L, left; R, right; A, anterior; P, posterior.

Time-series data for each subject, ROI and condition were concatenated into a 4D array (i.e., subject x time-point x ROI x condition). Then, correlations between all ROIs for each subject and condition were computed, taking time-points as individual observations in each ROI-to-ROI correlation. This resulted in three correlation matrices (i.e., one for each condition; Figure 3A) with three dimensions each (i.e., ROI x ROI x subject). Then, differences between the means of the correlation matrices, for each pair of conditions and across subjects, were computed for each ROI x ROI correlation matrix point (Figure 3B) and compared with the null-hypotheses distribution generated by randomly shuffling the condition labels over subjects and repeating this procedure for 1000 iterations (Figure 3C, α = 0.05, supra-threshold: in yellow). The resulting comparison matrices were corrected for multiple comparisons using pixel correction (Cohen, 2014; Figure 3C, pixel-corrected: in red). This procedure consists in picking the largest and smallest test statistic values of each permutation -which results in two distributions of extreme values- and then setting the thresholds for statistical significance to be the values corresponding to the 97.5 percentile of the largest value and the 2.5 percentile of the smallest value. Pixel correction was preferred as compared to the more common cluster-based methods because the nature of our data (i.e., correlation matrices) does not require that significant differences are spatially clustered.

#### Electric Current and Further Statistical Examinations

Furthermore, differences regarding the magnitude of the peak fields (99.9th percentile) between C1 and C2 were compared using a paired sample *t*-test. Additional paired sample *t*-tests were used to test for electric current magnitude differences between experimental conditions for each of the main ROIs selected based on rs-fMRI analyses. ROIs were selected and considered as main hubs when they involved ≥ 2 significant rs-FC couplings that survived multiple comparisons using pixel correction. Moreover, Pearson product-moment correlation was used to explore the association between tDCS-induced rs-fMRI effects and the magnitude of calculated current density estimates in the selected ROIs. More specifically, tDCS-induced functional changes in each particular coupling that survived pixel correction were linked to the selected ROIs’ electric current magnitude values when this fell within the rs-FC connection. Finally, to compare head movement and tDCS-related adverse events differences between the experimental groups, a one-way repeated measures ANOVA was used. In these analyses, data distribution was tested for normality with the Shapiro-Wilk test (*p* > 0.05; Shapiro and Wilk, 1965; Razali and Wah, 2011). Non-parametric tests were used in cases where parametric tests were not appropriate. These non-parametric tests are explicitly stated when necessary. No adjustment for multiple comparisons was applied in these exploratory statistical analyses. All these tests were two-tailed and α was set at 0.05.

## Results

### Multifocal Transcranial Direct Current Stimulation Effects on Resting-State Functional Magnetic Resonance Imaging

The two tDCS conditions differently influenced rs-fMRI connectivity. When comparing C1 against sham, some specific connections (i.e., a total of 10 resting-state couplings) significantly increased their coactivation (Figures 3D,F). Many of the affected connections involved temporal and temporo-occipital areas and distinct cerebellar regions. In particular, three temporal areas emerged as main hubs (i.e., those with ≥ 2 significant rs-FC couplings). These regions were the left and right temporo-occipital middle temporal gyri (toMTG.L and toMTG.R, respectively) and the right planum temporale (PT.R), which fall in the posterior part of the temporal lobe. Remarkably, C1 was also able to modulate structures entailing the limbic system, such as the amygdala and the hippocampal formation. When contrasting C1 against C2, two connections were detected to be significantly different (Figures 3E,G). These results represented occipital-cerebellar couplings. Particularly, we observed significant modification of the connections between the right lingual gyrus (LG.R) and the right supracalcarine cortex (SCC.R) and the seventh lobule of the vermis (Ver7). No differences were observed between C2 and sham.

### Electric Current Simulations

**FIGURE 4:**
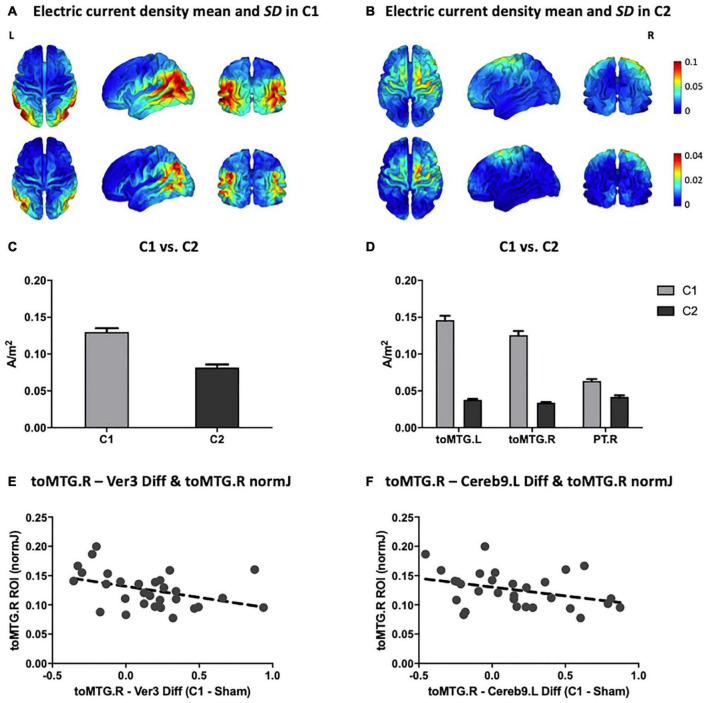
Multifocal tDCS simulated electric current distributions. Anatomical pattern of the electric current density mean (top) and *SD* (bottom) for all subjects in **(A)** C1 and **(B)** C2 (in A/m^2^ units). **(C)** Comparison between C1 and C2 of the mean magnitude of the peak fields (99.9th percentile; in A/m^2^ units). **(D)** Comparison between C1 and C2 of the electric current magnitude extracted from the three main hubs detected on the rs-fMRI analyses (i.e., toMTG.L, toMTG.R and PT.R; in A/m^2^ units). **(E,F)** Scatter plots showing the associations between multifocal tDCS-induced changes in rs-fMRI connectivity estimates and electric current magnitude values (in A/m^2^ units). Data in **(C,D)** are presented with mean with *SEM*. C1, condition 1; C2, condition 2; toMTG, Middle temporal gyrus, temporo-occipital part; PT, Planum temporale; Ver, Vermis; Cereb, Cerebellum; L, left; R, right; Diff, difference.

The means and *SDs* of electric current density distributions induced by C1 and C2 are displayed in Figure 4A (for C1) and Figure 4B (for C2). Individually modeled electric current density distributions are displayed in Supplementary Figure 1 (for C1) and Supplementary Figure 2 (for C2). The electric current density distribution induced by C1 predominantly included the inferior parietal lobule as well as temporal and occipital regions. In contrast, the electric current density induced by C2 showed a more anteriorly centered distribution principally encompassing the precentral, superior and, in a lesser extent, the middle frontal gyri. Moreover, as expected for the C2, the electric current magnitude within the posteromedial and occipital areas was very low. As can be further observed, the originally designed patterns created with the Colin27 template and the average simulated configurations obtained with our aged individuals were entirely anatomically consistent (see Figures 2E,F, 4A,B).

Furthermore, C1 reached statistically significant higher peak fields (99.9th percentile) as compared to C2 (*t* = 10.716; *p* < 0.001; Figure 4C). Additionally, the electric current magnitude values extracted from the three main ROIs identified on the rs-fMRI analyses (i.e., toMTG.L, toMTG.R and PT.R), were, as expected, significantly higher in C1 when compared to C2 (toMTG.L: *t* = 19.986, *p* < 0.001; toMTG.R: *t* = 18.473, *p* < 0.001; PT.R: Wilcoxon signed-rank test, *Z* = 4.000; *p* < 0.001; Figure 4D).

### Associations Between Resting-State Functional Magnetic Resonance Imaging Modulation and Induced Electric Current

Lastly, correlation analyses between tDCS-induced rs-fMRI changes and the magnitude of the calculated current density values were performed. We observed that those subjects who showed increased coactivation in C1 compared to sham in the toMTG.R – Ver3 coupling also presented lower induced electric current magnitude estimates in C1 (*r* = −0.401, *p* = 0.028; Figure 4E). In addition, those subjects who showed higher coactivation at C1 compared to sham in the toMTG.R – Cereb9.L also presented a negative association with the induced electric current magnitude values in C1, however, this association was not statistically significant (*r* = −0.358, *p* = 0.052; Figure 4F). Thus, it was observed that, although in the majority of cases (67.7% for the toMTG.R – Ver3 coupling; 64.5% for toMTG.R – Cereb9.L coupling) an increase in coactivation happened with certain levels of induced current, in those subjects with higher current density estimates, a functional coactivation reduction most likely occurred (see Figures 4E,F). Of note, since no significant differences were observed between C2 and sham, we focused these analyses to C1, which was significantly different from sham.

## Discussion

This represents, to the best of our knowledge, the first study investigating the impact of two distinct multifocal tDCS montages on rs-fMRI in healthy aging. Our results showed that: (I) multifocal tDCS modulates rs-FC in a montage-dependent manner in older adults. (II) Moreover, the functional impact is consistent with the spatial distribution of the induced electric current on the brain. (III) Finally, specific individual tDCS-induced rs-fMRI responses are related with the magnitude of the calculated electric current estimates, which might be in line with the stochastic resonance hypothesis (see below).

In this investigation, we observed that multifocal tDCS can modulate brain dynamics as measured through rs-fMRI connectivity amongst older adults. Furthermore, network-based tDCS appears to anatomically modulate functional connectivity in a manner dependent with the simulated electric current distribution. In this vein, the impact of transcranial stimulation was particularly evident when comparing C1 to sham. Three temporal regions emerged as the principal modulated regions: the left and right toMTG and the right PT. A relevant modulatory effect between these cortical nodes and the cerebellum was detected, though the modulation of cerebellar areas was less specific. Moreover, in other cortical temporo-parietal areas, such as the posterior divisions of the superior temporal and the supramarginal gyri, rs-FC was also modulated. In addition, the connectivity of subcortical regions, such as the amygdala and the hippocampal formation, which are core areas of the limbic system, was also modified. Of note, this system has been associated with emotion, motivation, and memory (Morgane et al., 2005), which are processes of particular significance in advancing age (i.e., Liu et al., 2020). In the present investigation, the main rs-fMRI results when contrasting C1 against sham (i.e., considering the main hubs; Figure 3F) topographically correspond with the estimated electric current distribution on the brain, as the largest electric current values in C1 were observed also in the inferior parietal and particular temporo-occipital brain regions (see Figure 4A). Furthermore, these results were consistent with the position of the electrodes that delivered the highest stimulation intensities in C1 (see Figure 2G and Table 1), namely P3 and P4 (with positive polarity), as well as P7 and P8 (with negative polarity). Interestingly, P3 and P4 electrode positions in the international 10-10 system are anatomically located above the inferior parietal lobule and principally encompass the Brodmann’s area 39 (BA39; Koessler et al., 2009). Moreover, the P7 and P8 electrodes are situated above the middle occipital and inferior temporal gyri and mainly include Brodmann’s areas 37 and 19, respectively (BA37 and BA19; Koessler et al., 2009). Hence, the highest intensity electrodes (regardless of the polarity) topographically covered the temporo-parieto-occipital junction (i.e., De Benedictis et al., 2014). In consequence, in the C1 montage, we observed, at the group level, a clear topographical association between the electrode’s assembly and injected current intensities, the corresponding calculated electric current density distribution in the cortical surface, and the neuroimaging results obtained with rs-fMRI data in our experimental design. Of note, no clear multifocal tDCS effects were observed at the rs-fMRI level in C2, potentially because the delivered stimulation was not sufficient to induce an observable functional connectivity modulation in the studied aging brains (for a visual inspection, compare Figures 4A,B, and see Figures 4C,D plots).

In previous literature, considerable inter-individual variability in response to distinct NIBS protocols has been observed (i.e., Hamada et al., 2013; López-Alonso et al., 2014; Wiethoff et al., 2014). These reports highlight the importance of identifying the individual predictors of NIBS effects, particularly in older adults, where substantial attempts have been made to modulate and optimize brain function and the associated cognitive performance (i.e., Antonenko et al., 2018; Nilakantan et al., 2019). In the present study we focused on the magnitude of the simulated electric current density as a potential factor contributing to such variability. In this vein, we investigated whether dissimilar individual differences in rs-fMRI modulation were related to the electric current magnitude estimates within the designated main ROIs. We observed that, in specific functional couplings, the higher the magnitude of calculated current values, the lower the coactivation increment. Thus, it is conceivable that aged individuals with dissimilar neuroanatomical characteristics (which relates to distinct simulated current density parameters) may require differential stimulation intensities to result in optimal modulations of functional brain connectivity. The hypothesis of the stochastic resonance might provide a mechanistic explanation of this state-dependency observation. According to Polanía et al. (2018), the stochastic resonance is a phenomenon referring to a situation in which a signal that is too weak to be detected by a sensor might be enhanced by adding an optimal level of noise. This assumption proposes that in a non-linear system, as the brain is, there are optimal noise levels for neural processing, and only intermediate, but not high or low levels of noise, can led to higher discriminability of the signal of interest. In this vein, the presence of neural noise (that can be added by means of NIBS) might confer to neurons more sensitivity to a given range of weak inputs, thereby rendering the signal stronger, or even synchronized (for a review, see Miniussi et al., 2013). This phenomenon has been reported in previous experimental NIBS studies, both using transcranial magnetic stimulation (TMS; Abrahamyan et al., 2011) and conventional tDCS (Peña-Gómez et al., 2011). More precisely, Abrahamyan et al. (2011) demonstrated that visual sensitivity can be improved with the right amounts of noise induced by means of TMS to the visual cortex. Further, in a previous study from our group, it was observed that distinct personality traits (introverts vs. extraverts), which might entail distinct baseline levels of brain activity, responded behaviorally different to the same tDCS protocol. The authors explained these results within the stochastic resonance paradigm, claiming that introverts, with higher levels of intrinsic neural activity, could have reached the threshold more easily than extraverts with a relatively weak electrical stimulation (see Peña-Gómez et al., 2011). Our data, using multifocal tDCS, appear to be consistent with the hypothesis that specific amounts of current density are required to produce an optimal neural effect (i.e., an increase in functional coactivation). On the contrary, both not delivering enough electrical current in the targeted brain structures (i.e., as could have occurred in C2), as well as a supplying supposedly excessive electrical current, could result in suboptimal neural effects (i.e., a functional coactivation reduction). In this vein, our results shows that the induced electric current magnitude estimates, which are contingent to the individual head and brain anatomy (Thielscher et al., 2011; Miranda et al., 2013), might entail a feasible predictive value regarding NIBS effects in aging when considered within this noise generation hypothesis.

Altogether, our data show that multifocal non-invasive stimulation models and protocols are capable to effectively modulate precise fMRI configurations in older adults. These observations may have important future implications for the cognitive neuroscience of aging field, since many of the neural basis regarding cognitive functioning, longitudinal trajectories and inter-individual differences, including the development of theoretical models, have been mainly based on studies employing this imaging technology (Grady, 2012; Cabeza et al., 2018; Vaqué-Alcázar et al., 2020). Furthermore, fMRI changes can effectively track the positive impact of behavioral interventions aimed to ameliorate cognition in the elderly (Duda and Sweet, 2020), including its combined effects with NIBS (Antonenko et al., 2018). Therefore, and as opposed to the use of conventional tDCS montages, the possibility of *a priori* designing particular NIBS-based interventions of regional-specific fMRI patterns may offer a valuable and refined approach for studies intended to optimize complex brain configurations amongst older individuals, or to investigate the impact of interventions in clinical trials. Moreover, the obtained tDCS results at the individual level are aligned with the notion that an exhaustive neuroanatomical characterization is critical to determine the right amount of stimulation required to produce optimal neurophysiological effects in each subject.

## Limitations

The present study is not without limitations. First, it is worth noting that the present investigation focused on the large-scale brain networks that hypothetically sustain cognitive aging, but not in cognitive performance itself. Hence, forthcoming studies should make an effort to unify the various levels of brain description (i.e., cells, networks, behavior) toward a comprehensive characterization of brain–behavior relationships in advanced age. Furthermore, although the observed results in fMRI data are unlikely to be due to poor control of the experimental conditions, our study could have improved methodologically by several different means, for instance, by using a sham condition for each real stimulation condition. Moreover, it is worth noting that our original modelings designed with Stimweaver were based on the template Colin27. Models based on aged brains or on individual MRIs could have improved the multifocal tDCS montage for both conditions. Furthermore, even though our individually generated models topographically matched the originally designed, future experimental investigations should incorporate both a T1- and a T2-weigthed acquisitions to optimize the electric current simulation procedures. Further in this line, it is pertinent for T1 images to be acquired without the stimulation cap assembled to avoid potential confounders in the segmentation process. Finally, it must be recognized that in all simulation-based electric current planning systems, there is uncertainty about the precise conductivity values that should be used for the different tissues and materials when creating and analyzing a model for a particular montage (see for further detail Miranda et al., 2013; Saturnino et al., 2019). Notwithstanding, notable advances are being achieved in this vein (Huang et al., 2017).

## Conclusion

Present results highlight that multifocal electrical stimulation protocols are capable of modulating neural dynamics in the elderly. Moreover, this functional modulation is related with the simulated electric current distribution on the brain. Thus, applying network-based procedures might entail a novel feasible approach to accurately target and modulate specific fMRI networks and connections critically involved in the cognitive aging process. Further, we have shown that the estimated magnitude of current density is a relevant factor accounting for the individual variability to NIBS in aged populations. Gathering knowledge about these variables will allow us to ultimately refine the parameters of transcranial stimulation to boost the brain and the potential cognitive benefits derived from NIBS-based interventions in advanced age.

## Data Availability Statement

The raw data and data processing code supporting the conclusions of this article will be made available by the authors, without undue reservation.

## Ethics Statement

The studies involving human participants were reviewed and approved by the Ethics Committee of the University of Barcelona (IRB 00003099). The patients/participants provided their written informed consent to participate in this study.

## Author Contributions

KA-P, LV-A, and DB-F designed and conducted the study and prepared the manuscript draft with important intellectual input from RP-A, CS-P, NB, RS, GR, MN, and AP-L. KA-P and LV-A completed the data collection. KA-P, LV-A, and RP-A performed the data analysis. All authors reviewed the manuscript.

## Conflict of Interest

RS and GR works for Neuroelectrics. MN serves on the scientific advisory board for Neuroelectrics and NeuroDevice. AP-L serves on the scientific advisory boards for Starlab Neuroscience, Neuroelectrics, Axilum Robotics, Constant Therapy, NovaVision, Cognito, Magstim, Nexstim and Neosync, and is listed as an inventor on several issued and pending patents on the real-time integration of transcranial magnetic stimulation with electroencephalography and magnetic resonance imaging. The remaining authors declare that the research was conducted in the absence of any commercial or financial relationships that could be construed as a potential conflict of interest.

## Publisher’s Note

All claims expressed in this article are solely those of the authors and do not necessarily represent those of their affiliated organizations, or those of the publisher, the editors and the reviewers. Any product that may be evaluated in this article, or claim that may be made by its manufacturer, is not guaranteed or endorsed by the publisher.

## Footnotes

1
http://fsl.fmrib.ox.ac.uk/fsl/fslwiki/

2
https://afni.nimh.nih.gov/

3
www.simnibs.org
