# Supplementary material for: Multifocal Transcranial Direct Current Stimulation Modulates Resting-State Functional Connectivity in Older Adults Depending on the Induced Current Density
Source: Front Aging Neurosci. 2021 Nov 26;13:725013. doi: 10.3389/fnagi.2021.725013 (PMC8662695; doi:10.3389/fnagi.2021.725013)
Supplement: Supplementary file 1 [file Data_Sheet_1.docx]

***Supplementary Material***

**Materials and Methods**

Experimental design: tDCS-related adverse events

Following previous procedures from our group (Abellaneda-Pérez et al., 2020), at the end of each experimental session, a questionnaire of tDCS-related adverse events was administered [adapted from Brunoni et al. (2011)]. All participants were asked if any tDCS-related adverse event was experienced both during as well as after the tDCS-MRI session. The adverse events studied were headache, neck pain, pain in the scalp, tingling, itching, burning sensation, local erythema, drowsiness, difficulty in concentration, and sharp mood swings. All the adverse events were classified as absent [0], mild [1], moderate [2] or severe [3]. Of note, as local erythema could not be detected during the tDCS-MRI session, this factor was not considered in the exploration of the adverse effects potentially occurring during the tDCS-MRI session.

In our sample, no significant differences were found between the three conditions, both considering the sum of adverse effects occurred during as well as after the tDCS-MRI experimental sessions (see Table S1).

| **TDCS-related adverse events** | **C1 (mean ± *SD;* median)** | **C2 (mean ± *SD;* median)** | **Sham (mean ± *SD;* median)** | **Friedman’s Test (*χ^2^, p*)** |
| --- | --- | --- | --- | --- |
| **During tDCS-MRI** | 0.774±1.407; 0 | 1.129±1.310; 1 | 1.419±1.858; 0 | *χ^2^*=2.519,  *p*=0.284 |
| **After tDCS-MRI** | 0.516±0.851; 0 | 0.548±1.207; 0 | 0.871±1.204; 0 | *χ^2^*=2.545,  *p*=0.280 |

**Table S1.** TDCS-related adverse events metrics both during and after tDCS-MRI sessions considering the three experimental conditions (C1, C2, and sham).

Experimental design: Quality of sham

At the end of the last experimental session, a very brief questionnaire about the quality of sham was administered. In this assessment, all participants were asked in which experimental session they conjectured to be under a placebo condition. The possible responses to this question were classified as in the first session [1], in the second session [2], in the third session [3], I do not know [4]. Furthermore, whether subjects selected a particular experimental session, they were asked how confident they were with the given response. The responses were classified from 1 to 5, being 1 not very convinced, and 5 very much convinced.

Data from the quality of sham questionnaire, that was answered by 30 participants, revealed that 13 subjects (43.3%) marked the option “I do not know”, 9 subjects (30.0%) correctly identified the sham session, and 8 subjects (26.7%) wrongly selected the sham session. Moreover, from the 17 participants that selected a particular experimental day as being the sham session, we obtained confidence estimations from 16 subjects. From the 8 subjects that correctly identified the sham session, 1 selected the “option 2” (12.5%), 5 selected the “option 3” (62.5%), and 2 selected the “option 4” (25%). From the 8 subjects that wrongly selected the sham session, 1 selected the “option 1” (12.5%), 1 selected the “option 2” (12.5%), 1 selected the “option 3” (12.5%), 4 selected the “option 4” (50.0%), and 1 selected the “option 5” (12.5%).

Functional connectivity preprocessing: Head movement

| **Head movement  measure** | **C1 (mean ± *SD;* median)** | **C2 (mean ± *SD;* median)** | **Sham (mean ± *SD;* median)** | **Friedman’s Test (*χ^2^, p*)** |
| --- | --- | --- | --- | --- |
| **Absolute  displacement** | 0.363±0.303; 0.289 | 0.340±0.159; 0.290 | 0.377±0.166; 0.357 | *χ^2^*=1.806,  *p*=0.405 |
| **Relative  displacement** | 0.081±0.041; 0.075 | 0.072±0.033; 0.069 | 0.074±0.039; 0.067 | *χ^2^*=2.323,  *p*=0.313 |

**Table S2.** Head movement estimates for both absolute and relative displacement considering the three experimental conditions (C1, C2, and sham).

**Results**

Electric current simulations: Individually modeled electric current density distributions

**Fig. S1.** Axial view of the simulated electric current density distributions computed in each subject in C1 (in A/m^2^ units). The # represents the identification of the thirty-seven subjects initially reached to participate in this study in correlative order. Note that these analyses were conducted on thirty out of the thirty-seven subjects originally contacted.

**Fig. S2.** Axial view of the simulated electric current density distributions computed in each subject in C2 (in A/m^2^ units). The # represents the identification of the thirty-seven subjects initially reached to participate in this study in correlative order. Note that these analyses were conducted on thirty out of the thirty-seven subjects originally contacted.

**References**

Abellaneda-Pérez K, Vaqué-Alcázar L, Perellón-Alfonso R, et al. Differential tDCS and tACS Effects on Working Memory-Related Neural Activity and Resting-State Connectivity. *Front Neurosci.* 2020;13:1440. doi:10.3389/fnins.2019.01440

Brunoni AR, Amadera J, Berbel B, Volz MS, Rizzerio BG, Fregni F. A systematic review on reporting and assessment of adverse effects associated with transcranial direct current stimulation. *Int J Neuropsychopharmacol.* 2011;14(8):1133-1145. doi:10.1017/S1461145710001690
